# On Modern Progress in Ophthalmic Medicine and Surgery

**Published:** 1896-03-07

**Authors:** Robert Brudenell Carter

**Affiliations:** Consulting Ophthalmic Surgeon to St. George's Hospital.


### On Modern Progress in Ophthalmic Medicine and Surgery.

By Robert Brudenell Carter, F.R.C.S., Consulting Ophthalmic Surgeon to St. George's Hospital.

LATENT SQUINT (*continued from p. 341*).

As long ago as in 1869, in the preface to my translation of Professor Scheffler's "Theorie der Augenfehler und der Brille," I called attention to the occasional failure of spectacles to relieve the conditions for which they were often successfully prescribed, saying that "cases every now and then present themselves in which there is manifestly present some unknown quantity, some disturbing factor, which the received doctrines do not take into account, and under which spectacles which seemed to be properly adapted to the requirements of the eyes are found to become sources of discomfort." Scheffler himself endeavoured to trace such results as these to neglect of the possible variations of convergence and divergence; and the investigations of subsequent American writers have included an upward or downward tendency of either eye as among the conditions which it is necessary to take into account. The main hypothesis on which their work in this direction has been founded is that the two eyes may have a tendency to a resting position in which their images could not be combined into a single one, and that, in all use, it becomes necessary to overcome this tendency by a muscular effort. They further maintain that, on account of the comparative weakness of the superior and inferior muscles, it is more difficult to overcome an upward or downward tendency than a tendency to convergence or to divergence.

The readiest and most simple means of ascertaining the resting position of the eyes is by using a little instrument called a "phorometer," which consists of a piece of glass, about three-quarters of an inch square, and convexly fluted, mounted in the centre of a disc of metal which can be dropped into the groove of a trial frame, either with or without a lens. A point of light, seen through this glass, assumes the appearance of a fine line, in a direction at right angles to the fluting. The patient is placed at a distance of from eight to twenty feet from the point of light, wearing a trial frame, with an opaque disc over the left eye. He is then made to look at the point of light with the right eye, and the phorometer is placed in the frame, with its fluting horizontal, so as to convert the point into a vertical line. The left eye should then be uncovered, so that with this the point will be visible under its natural appearance. If the eyes are both directed to the point, the line will appear to bisect it. If the eyes are unduly convergent, the line will be to the right of the point, and if they are unduly divergent, the line will be to the left of the point, the complete dissimilarity of the two images preventing any endeavour to combine them unless they fall naturally together. If the eyes are relatively convergent, and the line is to the right, a prism with its base outwards is held before the phorometer until one is found of a strength which carries the line through the point and measures the degree of the convergence. To measure relative divergence, the prism is held with its base inwards, and in either case the amount is expressed in angular degrees. When this examination is completed, the phorometer should be turned so as to render its flutings vertical, and the apparent extension of the point of light horizontal. The relative positions of line and spot will then show whether the eyes are coincident on a horizontal plane, or whether one is directed to a higher level than its fellow; and, in this case also, a prism is used to measure any deviation that may be found.

The importance which is attached to these deviations in America has now for some years been very considerable; and it is maintained by many surgeons that they furnish a clue by which nearly every case of asthenopia may be relieved; the means employed being either tenotomy of an over-active muscle, or advancement of the tendon of a weak one. Some of those who have written most upon the subject already count their operations of this class by hundreds, and speak of the proceeding as if it were one of the greatest triumphs of modern surgery. The trustworthy evidence, so far as I have been able to obtain access to it, falls far short of supporting the claims which have been made. The conditions of life in the United States are very favourable to the development of various neuroses, among which a real or fancied incapacity to use the eyes is common; and it is characteristic of many forms of neurotic ailment that the sufferers are apt to be relieved or cured, at least for a time, by any form of treatment which strongly impresses the imagination. The idea of "heterophoria" is simple and easily grasped, the explanation of the effect of an operation in overcoming it is simple and plausible, and the patient who has undergone tenotomy rather shrinks from the confession that no particular benefit has been derived from it. But, even in America, the operations have now fallen into some degree of discredit; and, at this very time, a committee appointed by the American Ophthalmological Society is engaged in an endeavour to distinguish facts from statements.

I have now, for a considerable period, examined the resting position of the eyes with the phorometer in every refraction case that has come before me. My experience is that some degree of heterophoria is the rule rather than the exception, and that its existence expresses nothing more than a certain degree of relaxation, of "standing easy," so to speak, of the muscles involved, at times when the attention is not actively directed to any visual impression. In ordinary cases the muscles spring promptly to "attention" at the word of command, and are so maintained without difficulty or conscious fatigue. In exceptional cases, either because the heterophoria is of large degree, or because the muscles are abnormally weak, the effort of overcoming it becomes at first irksome and at last impossible, and in these cases the surgeon may judiciously intervene. I have operated in several cases, in which there was incapacity to use the eyes, coupled with headache and general disturbance of the health, and in which the line and dot could not be united, on the vertical plane, by a prism of less than five degrees. In these cases the results have been generally satisfactory, but they need to be still further tested by time. So far I have not yet seen a case requiring operation in which the deviation was on the horizontal plane.

